# Supplementary material for: TRIM34 restricts HIV-1 and SIV capsids in a TRIM5α-dependent manner
Source: PLoS Pathog. 2020 Apr 13;16(4):e1008507. doi: 10.1371/journal.ppat.1008507 (PMC7179944; doi:10.1371/journal.ppat.1008507)
Supplement: Attachment — Submitted filename: Ohainle et al 2019 PLOS Pathogens.docx [file ppat.1008507.s002.docx]

Ohainle et al. present interesting findings that provide some mechanistic insight into how TRIM34 inhibits HIV replication. While TRIM34 has previously been studyied in the context of lentiviruses, the authors show here that TRIM34 function requires TRIM5alpha. Furthermore, the authors take advantage of their HIV-CRISPR screening tool to generate these novel results in an unbiased manner. Overall, I feel that this manuscript is appropriate for PLOS Pathogens granted that some revisions are done.

Major issues:

1. The authors should check to see how P90 affects restriction by TRIM34 in their ectopic TRIM34 expression system (Figure 2D).
2. Authors should perform the experiments in Figures 4A and 4B in the presence and absence of IFN (maybe they already have this?)
3. One noticeable absence from this paper is the study of non-human primate TRIM34. The authors indicate that TRIM34 is not rapidly-evolving in primates, but have they checked for overlapping function with human TRIM34? The authors have an experimental system in place to study ectopic TRIM34, so this would be an easy experiment to include. This would be a good addition to the paper because previous studies have already highlighted a role for TRIM34 in HIV restriction.

Minor issues:

1. Author summary: viruses don’t adapt to host *genes*, but rather host proteins. And HIV-1 does not interact with human *genes* (at least not in the sense that the authors are trying to describe). HIV-1 interacts with gene products (proteins).
2. Line 119: remove “are enriched in the viral supernatant.”
3. Fonts used to label different parts of Figure panels, including the titles, should be identical (I suggest Arial). For example, in Figure 2, “control” and “TRIM34” are Calibri, or something like that. This appears to be a common problem throughout all figures.
4. Figure 2: raw data (%GFP+ cells) makes an appearance in Figure 2. I suggest that some raw data is added to Figure 1 such that the effect of STAT, IFNAR, IRF9, and TRIM34 KO can be seen. It would also be nice to see the extent to which IFN inhibits LAI infection in THP1 here.
5. Can the authors discuss why TRIM34 KO rescues WT and N74D virus to nearly similar extents in the absence of IFN?
6. Line 187: what do the authors mean by a “knockout allele?”
7. Line 230: this statement is confusing. Actually, due to ongoing genetic conflicts between host and pathogens, restriction factors from one species *may not* restrict viruses adapted to other species (because virus capsids co-evolve with host-specific capsid-targeting factors). I suggest removing this line entirely as it is not required to ask whether TRIM34 acts broadly.
8. It is interesting to show and discuss the data in Figure 1 featuring both P90A and N74D. And the results from that Figure 1 justify a focus on N74D in subsequent figures. However, I would remove mention of P90A from the abstract as it is not the main focus of the paper and a recent paper has characterized the relationship between TRIM5 and this CypA binding deficient mutant.
9. I think that more discussion is warranted on the following topic: does TRIM5 need TRIM34 in order to restrict HIV (WT or N74D)? Why or why not? The authors only focus the discussion on how TRIM34 needs TRIM5alpha in order to restrict N74D. The title states how “TRIM34 works with TRIM5.” This word choice is vague, and it begs the question of whether some or all activities of TRIM5 are dependent on TRIM34. This title may also give the impression that TRIM34 and TRIM5 work in an additive (independent) fashion. An alternative title could be: “TRIM34 restricts HIV and SIV capsids in a TRIM5-dependent manner.”
10. The authors should include a mention of the data that TRIM34 has different specificity than TRIM5 (it inhibits SIVmac) in the Discussion. Can they speculate as to why TRIM34+TRIM5 may restrict SIVmac CA but TRIM5 alone does not?
11. Line 386: the language used here is confusing. Do references 43 and 44 conflict with one another? Does N74D exhibit slower uncoating kinetics in one paper but not the other? Similarly, at line 394, is it implied that N74D uncoats and transits more slowly than WT? Please clarify.
