# Supplementary material for: TRIM34 restricts HIV-1 and SIV capsids in a TRIM5α-dependent manner
Source: PLoS Pathog. 2020 Apr 13;16(4):e1008507. doi: 10.1371/journal.ppat.1008507 (PMC7179944; doi:10.1371/journal.ppat.1008507)
Supplement: Attachment — Submitted filename: 2020_03_23 Ohainle response to reviewers.docx [file ppat.1008507.s003.docx]

We thank the reviewers for their careful analysis of our study. As described below the manuscript has been significantly strengthened through both the inclusion of additional experiments and through improved communication of results. We have added clarification to areas of unclear explanations of the results and further have improved interpretations of the results as well as included additional key discussion points in large part thanks to the reviewer’s comments.

**Part II – Major Issues: Key Experiments Required for Acceptance**

Reviewer #1: (No Response)

Reviewer #2:
1. The authors should check to see how P90 affects restriction by TRIM34 in their ectopic TRIM34 expression system (Figure 2D).

Response: We have addressed the role of TRIM34 on the P90A mutant using our gene knockout cells, which is a better experiment to determine the role of physiological levels of TRIM34 than the over-expression assays. Our data in primary CD4+ T cells knocked out for TRIM34 or TRIM5 (Figure 4C) shows that TRIM5 is the dominant restriction to the P90A, but that TRIM34 knockout had no effect. In MoDCs (Figure 4F) in which we show that TRIM5 is also the dominant restriction factor, we do see a small increase in virus levels when TRIM34 is knocked down (2.3-Fold Rescue). These data are consistent with the data from the HIV-CRISPR screen that we show in Figure 1B for THP-1 cells where TRIM34 does score higher in the P90A screen than in the WT screen, albeit significantly lower than TRIM5. We now more specifically point this out in these results in the text (Lines 151-154).

2. Authors should perform the experiments in Figures 4A and 4B in the presence and absence of IFN (maybe they already have this?)

Response: We were not clear enough in the paper about experiments that were done in either the presence and absence of IFN. We believe this confusion stems from an inadequate explanation on our part of the IFN-independence of TRIM34’s restriction (a key point that was also brought up by other reviewers). We show in Figure 2A and 2B that the effect of TRIM34 is independent of IFN treatment and instead is a constitutively-expressed restriction factor. However, we agree that this point was not sufficiently explained and we have now modified text to highlight the key fact that while the screen was performed in IFN-treated cells, TRIM34 restriction is observed in the absence of IFN (Lines 193 – 196). To further highlight this key point we have moved the data showing validation of TRIM34’s constitutive effects through screening in cells without any IFN treatment into Figure 2 (Figure 2C) from the supplemental data (Figure S1).

3. One noticeable absence from this paper is the study of non-human primate TRIM34. The authors indicate that TRIM34 is not rapidly-evolving in primates, but have they checked for overlapping function with human TRIM34? The authors have an experimental system in place to study ectopic TRIM34, so this would be an easy experiment to include. This would be a good addition to the paper because previous studies have already highlighted a role for TRIM34 in HIV restriction.

Response: We have now done this experiment. We now include data showing that rhesus macaque TRIM34 phenocopies human TRIM34 in restricting the N74D mutation (Figure 3F and 3G). Text describing this data is now included in the results (Lines 278-289) and discussion (Lines 417-425). These data are consistent with the idea that *TRIM34* gene is not under positive selection, but that the specificity for the restriction may come from interaction with TRIM5. Future experiments with additional TRIM5 and TRIM34 pairs from different primates are being planned as a more extensive follow up to this hypothesis.

Reviewer #3:

1. The authors state that loss of CPSF6 binding is not sufficient for TRIM34 restriction as A77V HIV-1 is not restricted. However, this is an unusual mutant that is not restricted in general in macrophages. It would more convincing for the authors to evaluate other capsid mutants that do not bind to CPSF6 and are restricted in primary cells (i.e. N57A/S and/or A105T). Also, does TRIM34 restrict non-primate lentiviruses (i.e. FIV and EIAV)?

Response: This was a good idea. As the reviewer points out, the phenotype of the A77V mutant differs significantly from the N74D mutant in some cell types. We have now included data from the N57A capsid mutants to complement our findings with the N74D and A77V capsid mutants (Figure 3C). We find that N57A, like A77V, is not sensitive to TRIM34 and this data has been added to the results and included in the discussion (Lines 401 – 402). Human TRIM34 has previously been shown to have modest restriction activity against EIAV but not N-MLV and this information is now included in the discussion (Lines 372 – 373). We have also expanded the discussion to include the observation that N74 is highly conserved in primate lentiviruses and, therefore, escape from TRIM34 restriction may represent a significant cellular barrier for primate lentiviruses (Lines 417 – 425).

2. The authors initially identified TRIM34 restriction of N74D HIV-1 in IFN-treated THP-1 cells and state in Lines 322-325: "Our HIV-CRISPR screen demonstrates that the increased IFN-sensitivity of the HIV-1 N74D CA mutant [19] is not due to an increased sensitivity to the known capsid-targeting restriction factors TRIM5α or MxB (Figure 1B) but rather is due to restriction by a novel capsid-targeting restriction factor, TRIM34." However, other than this initial screen described in Figure 1 and data shown in Figure 2A, the remainder of the manuscript does not appear to include IFN-treated cells. In Figure 2, the level of restriction with or without IFN treatment is similar (2.2-fold vs. 2.0-fold, respectively). It is unclear how TRIM34 enhances IFN sensitivity, as this is not shown in the presented data.

Response: The reviewer is correct that this phrasing is misleading. We have removed this sentence and instead have added text to explain more clearly that TRIM34 could be important either in IFN-mediated restriction or that it could be IFN-independent (Lines 193-196). As the reviewer points out the remaining experiments are done without IFN as we show that the effect of TRIM34 occurs independent of IFN. We have emphasized this by 1) including screen data performed without IFN in Figure 2 (Figure 2C) and by 2) rewording the discussion to put less emphasis on the role of TRIM34 on IFN inhibition (Lines 369-370).

3. CypA is discussed throughout the manuscript as being an important capsid-binding factor. Yet other than evaluation of P90A HIV-1 (and SIV), CypA is not directly addressed. This is surprising as 1) some of the co-authors of this manuscript recently published that CypA protects HIV-1 from TRIM5α and 2) Ambrose et al. showed that N74D HIV-1 is more sensitive to loss of CypA binding than WT HIV-1. Is TRIM34 restriction of N74D HIV-1 influenced by CypA?

Response: The reviewer is correct that there is potentially interesting interplay between different capsid-targeting restriction factors and various capsid mutant viruses. While we agree that there is potential interplay between TRIMs and cyclophilin-dependence/independence we believe this to be outside the scope of this study.

4. There are some concerns regarding the imaging experiments in Figure 5. First, the data represent staining at 2h post-infection, which would be significantly later than capsid uncoating. Do the authors anticipate earlier binding of TRIM34/TRIM5α to N74D CA? Second, do the HeLa cells that express tagged TRIM34 and TRIM5α restrict N74D HIV-1? Finally, colocalization of N74D CA is higher with TRIM34 with half of them not associated with TRIM5α. Does this suggest a sequential interaction of the 2 host proteins with N74D capsid?

Response: Although we appreciate that many views of uncoating and its associated kinetics exist in the field, we would respectfully disagree that uncoating has completed at this time. While the spatiotemporal staging of uncoating remains a somewhat contentious topic, we would respectfully offer the following:

1) A very popular model of uncoating posits that the viral core must remain intact until the time of nuclear import to prevent the viral genome from being sensed by cytoplasmic nucleic acid sensors such as cGAS (Campbell and Hope, 2015; Hilditch and Towers, 2014). Data supporting such a model would not be consistent with uncoating being completed in this time frame. A recent paper from the Melikian group observed that uncoating that led to infection could be visualized at the NPC 4-8 hours PI (See Fig 3 in (Francis and Melikyan, 2018)).
2) While there is currently little consensus on when uncoating is complete, or even what the completion of uncoating means, we would argue that uncoating is complete when assembled CA (the target of TRIM5 and TRIM34) no longer plays a functional role in infection. The compound PF74 binds specifically to assembled CA and is known to inhibit infection. HIV-1 infection remains susceptible to inhibition by PF74 8 or more hours after infection (Fig 3 in (Saito et al., 2016)).

We therefore think there is reasonable evidence to support the premise that uncoating is not complete 2 hours after infection. Finally, we would add that the 2 hour time point was chosen because this was the time point which most effectively allowed HIV-1/rhTRIM5a interactions to be visualized in our previous work (Fig 2 in (Campbell et al., 2008)).

Second, yes we do observe some restriction of the N74D virus in the cells used for colocalization experiments. That data is included below. We have not added this to the manuscript as we feel this is a minor point and this system (in which these HeLa cells still express endogenous levels of TRIM5 and TRIM34) is not ideal.

Third, we thank the reviewer for the observation that some N74D capsids are colocalizing with TRIM34 independent of TRIM5 and this could, as the reviewer points out, be due to sequential binding of TRIMs. One of the most variable regions between TRIM5 and TRIM34 is the capsid-binding PRY/SPRY domain, so we believe that it is indeed possible that mechanisms of capsid binding could differ significantly between these two orthologues. However, it is important to note that in the system we are using here, these HeLa cells express endogenous levels of TRIM5 and, therefore, we cannot conclude that TRIM34 is binding N74D capsids independent of TRIM5.

**Part III – Minor Issues: Editorial and Data Presentation Modifications**

Reviewer #1: The TRIM34 restriction is only seen in lab generated mutants of HIV-1 capsid and not wild type. Unless these mutations occur naturally, the title claiming that TRIM34 restricts HIV-1 is a little misleading. Perhaps “primate lentiviral capsids” will be more appropriate.

Response: We appreciate the reviewer’s suggestion. However, we would like to maintain both HIV and SIV in the title as the restriction is, in fact, a restriction of an HIV virus. Yes, it has a virus with a capsid mutation but we believe the restriction of HIV capsids with single amino acid mutations is important. For example, this data suggests that TRIM34 may limit the evolutionary pathways available to HIV capsids in human cells.

Given that the restriction is stronger against the SIVs, it would seem that TRIM34 could work with TRIM5α to prevent cross-species transmission of primate lentiviruses, making the SIV results as important, if not more, than the weak effects seen with the artificial HIV-1 mutants. The authors have hinted at this in the penultimate paragraph on page 11 but the point could be emphasised more strongly.

Response: We agree that the significant restriction of SIV is an important finding. We have added more text to further highlight the significance of this finding to the discussion (Lines 417-425).

TRIM34 scored as highly as the IFN pathway genes in the HIV-CRISPR screen. Yet, its effects are weak in subsequent experiments. Given that the readout of the HIV-CRISPR screen is from viruses that are released from infected cells, this would identify processes from early as well as late stages of the life cycle. However, the subsequent experiments measured percentage of infected cells after 48 hours, which is probably the result of a single cycle of infection, hence only early stages of the life cycle. If TRIM34 works with TRIM5α, which has been shown to also act as an immune sensor, the possibility that it could trigger the innate immune pathways cannot be discounted. This could activate genes that might act on the late stages of the viral life cycle and would not be picked up looking at infected cells from a single cycle infection. Indeed, N74D has been previously reported to trigger innate sensors due to lack of Cpsf6 binding. However, this will have to be reinterpreted in the light of the data from Fig 3A and 3B.

Response: The reviewer is correct that TRIM34’s effects are IFN-independent and, therefore, TRIM34 is not the IFN-induced factor we set out to find. This has been further clarified in the Results (Lines 193-196) and in the Discussion (Lines 369-370). Further we agree, as the reviewer has highlighted, that our work has implications for innate immune sensing mediated by TRIM5. While we have not directly addressed any role of TRIM34 in sensing pathways in this study, we have added a discussion of the potential for TRIM34 effects on TRIM5 sensing (Lines 498 – 503).

Other points:

Typos: Line 45 on page 2, “… the HIV-1 capsid protein is exposed to an array of …”

Response: corrected (Line 45).

Line 150 on page 5, “However, unlike T TRIM5α ,…” T shouldn’t be there.

Response: corrected (Line 160).

Fig 2A and 2B. The legend describes the assay as intracellular P24gag+ staining but the y-axis is labelled as %GFP+ cells. Is the readout p24gag+ or GFP+?

Response: The readout in this experiment is %GFP+ cells. The Figure legend has been corrected.

Fig 2C and Fig 3B. These are very similar experiments involving CD4+ T cells. However, the infectivities of WT and N74D viruses are different (5% GFP+ for WT, 1% GFP+ for N74D/TRIM34KO in Fig 2C; 1% GFP+ for WT, 4%GFP+ for N74D/TRIM34 KO in Fig3B). The effect of TRIM34KO also varies. (8x in Fig 2C and 4.2x in Fig 3B). Is it amount of input virus the same or normalized in anyway? The variation of 2-fold is of concern since some of the TRIM34 effects described (eg Fig 2A and 2B) are 2-fold.

Response: The reviewer has highlighted a point of confusion regarding infection levels across our experiments. We have now added more information to the Figure Legend (Figure 3) to more clearly highlight the fact that this data is shown as “relative infection” where each virus is normalized to the control infection. As the reviewer points out, we do observe variability in overall infection levels across CD4+ isolations in these experiments. For the experiments in CD4+ T cells, we believe that this level of variability is within what would be expected across different cell donors as well as different crRNP electroporations where the editing levels can also vary from experiment to experiment.

Fig 2E. The significance of the graphs showing Neviparin treatments were not described in the text.

Response: We thank the reviewer for finding this oversight. This information has now been added to the Results (Lines 229-231).

Reviewer #2: Minor issues:

Author summary: viruses don’t adapt to host *genes*, but rather host proteins. And HIV-1 does not interact with human *genes* (at least not in the sense that the authors are trying to describe). HIV-1 interacts with gene products (proteins).

Response: We thank the reviewer for the attention to detail. We have corrected this (Lines 43 and 51).

1. Line 119: remove “are enriched in the viral supernatant.”

Response: this has been deleted.

2. Fonts used to label different parts of Figure panels, including the titles, should be identical (I suggest Arial). For example, in Figure 2, “control” and “TRIM34” are Calibri, or something like that. This appears to be a common problem throughout all figures.

Response: Again, we thank the reviewer for their attention to detail and for the Font suggestion. We have made the Font used in Figure panels consistent throughout the manuscript.

3. Figure 2: raw data (%GFP+ cells) makes an appearance in Figure 2. I suggest that some raw data is added to Figure 1 such that the effect of STAT, IFNAR, IRF9, and TRIM34 KO can be seen. It would also be nice to see the extent to which IFN inhibits LAI infection in THP1 here.

Response: We thank the reviewer for these suggestions, but have not included this here as we believe data is most clearly presented in the current form. We have not included the IFN inhibition data as it has been published elsewhere by us and others (Bulli et al., 2016; OhAinle et al., 2018).

4. Can the authors discuss why TRIM34 KO rescues WT and N74D virus to nearly similar extents in the absence of IFN?

Response: As the other reviewers pointed it, our text was not clear that we observe a restriction by TRIM34 both in the presence and the absence of IFN. We now more specifically point that out in the context of Figure 2.

5. Line 187: what do the authors mean by a “knockout allele?”

Response: This phrase has now been removed to prevent confusion in terminology (Line 209).

6. Line 230: this statement is confusing. Actually, due to ongoing genetic conflicts between host and pathogens, restriction factors from one species *may not* restrict viruses adapted to other species (because virus capsids co-evolve with host-specific capsid-targeting factors). I suggest removing this line entirely as it is not required to ask whether TRIM34 acts broadly.

Response: We understand the confusion. However, we do believe that our logic in explaining this experiment is sound as it is a fundamental aspect of this work that viruses that are well-adapted to replicate in one species can be sensitive to restrictions from another species (e.g. see the work on A3G-Vif evolution so elegantly explored here: https://www.ncbi.nlm.nih.gov/pubmed/23359341). Therefore, we have left this sentence in the manuscript.

7. It is interesting to show and discuss the data in Figure 1 featuring both P90A and N74D. And the results from that Figure 1 justify a focus on N74D in subsequent figures. However, I would remove mention of P90A from the abstract as it is not the main focus of the paper and a recent paper has characterized the relationship between TRIM5 and this CypA binding deficient mutant.

Response: We have now removed P90A from the abstract.

8. I think that more discussion is warranted on the following topic: does TRIM5 need TRIM34 in order to restrict HIV (WT or N74D)? Why or why not? The authors only focus the discussion on how TRIM34 needs TRIM5alpha in order to restrict N74D. The title states how “TRIM34 works with TRIM5.” This word choice is vague, and it begs the question of whether some or all activities of TRIM5 are dependent on TRIM34. This title may also give the impression that TRIM34 and TRIM5 work in an additive (independent) fashion. An alternative title could be: “TRIM34 restricts HIV and SIV capsids in a TRIM5-dependent manner.”

Response: As the reviewer points out, the dependence of TRIM34 on TRIM5 is not necessarily symmetric (ie, TRIM5 doesn’t depend on TRIM34 in the same way). This is one of the most interesting questions moving forward in understanding TRIM34 and TRIM5 restriction. We thank the reviewer for the title suggestion and we have made this change.

9. The authors should include a mention of the data that TRIM34 has different specificity than TRIM5 (it inhibits SIVmac) in the Discussion. Can they speculate as to why TRIM34+TRIM5 may restrict SIVmac CA but TRIM5 alone does not?

Response: We thank the reviewer for highlighting this key point. We do observe restriction mediated by TRIM34 that is not observed with overexpression of TRIM5 alone for both the N74D capsid mutant virus and for SIVmac (see Lines 417-425 for additional discussion of this point).

10. Line 386: the language used here is confusing. Do references 43 and 44 conflict with one another? Does N74D exhibit slower uncoating kinetics in one paper but not the other? Similarly, at line 394, is it implied that N74D uncoats and transits more slowly than WT? Please clarify.

Response: As the reviewer points out this aspect of the discussion is confusing. We have now reorganized and rewritten this to make this logic more clear (Lines 461-474).

Reviewer #3:

1. Line 45: the word "an" should precede "array of capsid-targeting"

Response: corrected.

2. Lines 103-104: P90A is not CypA-deficient but rather CypA binding-deficient. Similarly, N74D is not CPSF6-deficient but deficient in binding to CPSF6.

Response: We thank the reviewer for catching this error and have corrected this (Lines 99-100).

3. Line 133: The preceding sentence describes "ISGs, including MXB (MX2), IFITM1, TETHERIN (BST2) and TRIM5." Only IFITM1 and TETHERIN is in cyan in Figure 1; TRIM5 and MXB are yellow and dark blue, respectively.

Response: yellow and dark blue has been added to the Results (Line 141) and to the Figure Legend.

4. Line 327: should be "G89V" instead of "G98V"

Response: corrected.

Other minor changes:

Figure 2, 3 and 4 – made Y-axis labels consistent (ie, “Relative Luciferase Units” instead of “RLU” and “relative infection (%GFP+ cells)” instead of “relative infectivity”). Updated Figure Legends accordingly.

Updated Figure 5B to a “****” to show statistical significance to match other Figures.

Increased size of Figure 5A to make punctae more visible.

Methods and Figure Legends updated to reflect new data.

Sequencing data from HIV-CRISPR screening in this paper has been deposited to the Gene Expression Omnibus and this information has been added to the methods.

An ethics statement has been added to the Materials and Methods (Lines 549-554).

An additional funding source has been added (P50 AI150464-13 to M.O.).

REFERENCES

Bulli, L., Apolonia, L., Kutzner, J., Pollpeter, D., Goujon, C., Herold, N., Schwarz, S.M., Giernat, Y., Keppler, O.T., Malim, M.H.*, et al.* (2016). Complex Interplay between HIV-1 Capsid and MX2-Independent Alpha Interferon-Induced Antiviral Factors. J Virol *90*, 7469-7480.

Campbell, E.M., and Hope, T.J. (2015). HIV-1 capsid: the multifaceted key player in HIV-1 infection. Nat Rev Microbiol *13*, 471-483.

Campbell, E.M., Perez, O., Anderson, J.L., and Hope, T.J. (2008). Visualization of a proteasome-independent intermediate during restriction of HIV-1 by rhesus TRIM5alpha. J Cell Biol *180*, 549-561.

Francis, A.C., and Melikyan, G.B. (2018). Single HIV-1 Imaging Reveals Progression of Infection through CA-Dependent Steps of Docking at the Nuclear Pore, Uncoating, and Nuclear Transport. Cell Host Microbe *23*, 536-548 e536.

Hilditch, L., and Towers, G.J. (2014). A model for cofactor use during HIV-1 reverse transcription and nuclear entry. Curr Opin Virol *4*, 32-36.

OhAinle, M., Helms, L., Vermeire, J., Roesch, F., Humes, D., Basom, R., Delrow, J.J., Overbaugh, J., and Emerman, M. (2018). A virus-packageable CRISPR screen identifies host factors mediating interferon inhibition of HIV. Elife *7*.

Saito, A., Ferhadian, D., Sowd, G.A., Serrao, E., Shi, J., Halambage, U.D., Teng, S., Soto, J., Siddiqui, M.A., Engelman, A.N.*, et al.* (2016). Roles of Capsid-Interacting Host Factors in Multimodal Inhibition of HIV-1 by PF74. J Virol *90*, 5808-5823.
